# Digital Health Applications for Pharmacogenetic Clinical Trials

**DOI:** 10.3390/genes11111261

**Published:** 2020-10-26

**Authors:** Hetanshi Naik, Latha Palaniappan, Euan A. Ashley, Stuart A. Scott

**Affiliations:** 1 Department of Genetics and Genomic Sciences, Icahn School of Medicine at Mount Sinai, New York, NY 10029, USA; sascott@standford.edu; 2 Department of Medicine, Stanford University Medical Center, Palo Alto, CA 94305, USA; lathap@stanford.edu; 3 Department of Medicine, Genetics, and Biomedical Data Science, Stanford University Medical Center, Palo Alto, CA 94305, USA; euan@stanford.edu; 4 Stanford Medicine Clinical Genomics Program, Stanford Health Care, Stanford, CA 94305, USA

**Keywords:** pharmacogenetics, clinical trials, digital health, health information technology, wearable devices, telehealth, personalized medicine

## Abstract

Digital health (DH) is the use of digital technologies and data analytics to understand health-related behaviors and enhance personalized clinical care. DH is increasingly being used in clinical trials, and an important field that could potentially benefit from incorporating DH into trial design is pharmacogenetics. Prospective pharmacogenetic trials typically compare a standard care arm to a pharmacogenetic-guided therapeutic arm. These trials often require large sample sizes, are challenging to recruit into, lack patient diversity, and can have complicated workflows to deliver therapeutic interventions to both investigators and patients. Importantly, the use of DH technologies could mitigate these challenges and improve pharmacogenetic trial design and operation. Some DH use cases include (1) automatic electronic health record-based patient screening and recruitment; (2) interactive websites for participant engagement; (3) home- and tele-health visits for patient convenience (e.g., samples for lab tests, physical exams, medication administration); (4) healthcare apps to collect patient-reported outcomes, adverse events and concomitant medications, and to deliver therapeutic information to patients; and (5) wearable devices to collect vital signs, electrocardiograms, sleep quality, and other discrete clinical variables. Given that pharmacogenetic trials are inherently challenging to conduct, future pharmacogenetic utility studies should consider implementing DH technologies and trial methodologies into their design and operation.

## 1. Introduction

Digital health (DH) broadly refers to the use of digital technologies and data analytics to understand health-related behaviors, which ultimately can be utilized to enable more personalized clinical care [1,2]. This includes mobile health applications, health information technology, wearable devices, and telehealth. Accordingly, DH is increasingly being used in clinical trials, with specific recommendations for its use being made by clinical trial networks for mobile data collection and management, protocol design, and other applications [3]. An emerging research area that could potentially benefit from incorporating DH is pharmacogenetic clinical trials. Pharmacogenetics has long been identified as an actionable subspecialty of personalized medicine; however, prospective trials with the primary goal of assessing pharmacogenetic-guided therapy have been difficult to conduct. 

Prospective pharmacogenetic trials usually compare a standard dosing arm (standard of care control) to a pharmacogenetic-guided arm, often involving a therapeutic algorithm. They are generally designed as single-blinded, where the participant is unaware of their arm, but the prescriber/investigator is aware, as blinding the prescriber presents many challenges when implementing medication adjustments, or if the pharmacogenetic-guided intervention is only a ‘recommendation’ to be tailored by physician judgement. Operationalizing randomized trials has also been a notable issue for pharmacogenetic studies. For example, although many clinical genotyping panels currently are available to guide antidepressant prescribing, to date there have only been five antidepressant pharmacogenetic trials reported that were randomized, all of which were single-blinded [4]. 

Prospective pharmacogenetic trials also often require large sample sizes (given modest effect size), and recruitment can be difficult as they frequently need to identify eligible patients before they are prescribed medications of interest. Other issues with pharmacogenetic trials include a lack of ethnic diversity, rare variants with large effect, environmental and clinical confounders, as well as logistical issues including complicated delivery of therapeutic recommendations to both investigators and patients (whether blinded or not), manual tracking of medication adverse events, and patient compliance [4,5]. However, the use of DH technologies offers significant potential for improving pharmacogenetic trial design, which ultimately may facilitate more efficient and cost-effective trial operations [6].

## 2. Traditional Clinical Trials

**Figure 1:**
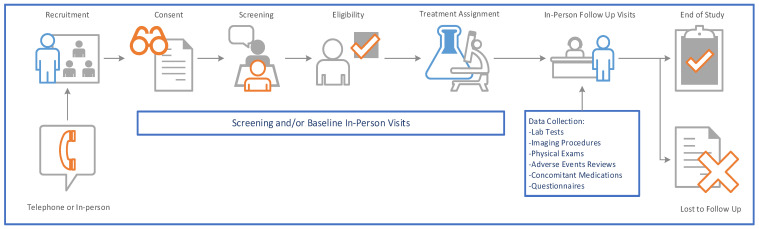
Traditional model of randomized controlled trials with data collected at in-person study visits.

Randomized controlled trials (RCTs) have always been considered the gold standard for determining the efficacy of an intervention. Generally, the main activities are conducted in-person at a clinical site, including the consent process and all data collection procedures (e.g., physical exams, administration of study drug, sample collection, tests such as imaging studies) (Figure 1). However, in-person study visits can be many and frequent, requiring a significant time commitment from the patient, study investigators, and other research personnel (e.g., coordinators, research nurses, pharmacists). Moreover, traditional RCTs historically necessitate considerable effort from trial sponsors and contract research organizations.

Conducting traditional pharmacogenetic RCTs can be challenging to operationalize and implement [7]. For example, RCTs can have extensive inclusion and exclusion criteria (IEC), which limit patient enrollment and can lead to trial results with limited generalizability. This can be particularly problematic in a pharmacogenetic trial where studying ethnically under-represented patient populations has been noted as an unmet need in the pharmacogenetics field [8,9].

Given these challenges with implementing RCTs for pharmacogenetic interventions, pragmatic clinical trial designs have increasingly been pursued as an alternative utility study that is complementary to the traditional RCTs [10]. By definition, pragmatic clinical trials focus on treatments and outcomes in ‘real-world’ health system environments rather than focusing on proving causative explanations for outcomes as in RCTs [11,12]. Pragmatic trials generally have broader IEC, meant to be more like real world patient populations, and therefore these trials are designed to be more generalizable. Pragmatic trials can provide useful data for pharmacogenetic studies looking to assess real world data, and using DH technologies to capture this data could simplify study conduct. Subsequent sections in this review present use cases of DH technologies that could be applied to pharmacogenetic trials in an effort to improve trial design, lower costs, increase patient engagement, and measure outcomes. 

## 3. Current Applications of Digital Health in Clinical Research

DH technologies have already had a wide range of uses in clinical research and trial implementation. For example, wearable devices have collected biometric outcome data in cardiac studies [13,14], smartphone apps have collected sleep quality data [15], electronic screeners have resulted in more accurate reporting of risk behavior (e.g., assessments completed by a patient on a tablet), including substance use [16], and a text messaging intervention has improved adherence to stimulants among children and adults with ADHD [17,18]. Although new wearable devices and apps always require validation and testing prior to implementing in clinical research studies, these DH tools are promising advances that can help operationalize innovative trials. DH interventions also have the potential to help overcome some of the health disparities in treatment access and lack of diversity commonly observed in many trials [19].

## 4. Digital Health Use Cases in Pharmacogenetic Trials

### 4.1. Electronic Consenting (E-Consenting)

E-Consenting refers to using electronic systems and processes to communicate information related to the study and to obtain and document informed consent from a participant [20]. E-consenting can range from being completely automated and include pre-recorded information, graphics, images, etc., to including some interaction with the study team, or being contacted by the study team remotely via telehealth and using electronic signing systems for consenting. Of note, the Food and Drug Administration (FDA) provides important guidance on electronic signatures (21 CFR Part 11) [21]. Pharmacogenetic trials are generally complex and typically involve medication and/or dosing changes. As such, e-consenting for pharmacogenetic trials likely could be best served by a hybrid model of automated content and abbreviated interaction with the study team via telehealth.

### 4.2. Electronic Health Record (EHR) Enabled Enrollment

Patient recruitment is difficult for most clinical trials [22], and pharmacogenetic trials are no exception. Targeted recruitment with EHR data can help identify potentially eligible subjects (sometimes referred to as ‘clinical trial recruitment support systems’) by screening for potentially eligible patients (by age, disease state, race/ethnicity, etc.). It can be an effective tool to identify patients that study personnel can follow up with to conduct a more thorough screen if necessary. Though EHR targeted recruitment is not standard, it has been used in some cases with success [23]. Pharmacogenetic trials in particular could benefit from digital screening to identify patient populations that are more likely to be prescribed medications of interest based on predefined demographic and clinical variable algorithms.

### 4.3. Patient-Reported Outcomes (PROs) and Health Applications (Apps)

Patient-reported outcomes (PROs) are increasingly being recognized as important endpoints in trials, but collecting them can be burdensome as they are often derived from paper questionnaires (or more recently administered on iPads or tablets), and are given frequently during the course of a trial. Some trials may even require daily diaries to be completed [24]. Having these administered via an app on a patient’s smartphone is preferable to administering the tools in-person, over the phone, or even on a separate device given to the patient. Apps can also be used to increase patient engagement, for remote symptom monitoring and collection of adverse events (AEs) in real time, and for digital phenotyping [25]. A notable AE that could be easily tracked by an app for pharmacogenetic trials is assessing the frequency and severity of skin reactions. This could prevent patients from having to present to a site for milder reactions (e.g., atopic dermatitis).

Digital mental health apps are one of the most downloaded health apps [26], and psychiatric/antidepressant pharmacogenetic trials should likely consider partnering for collection of PRO data. More innovative methods can be employed as well to decrease study personnel involvement, including an algorithm of reporting AEs to chat bots that can trigger the scheduling of a telehealth visit with an investigator. In addition, web-based therapeutic education systems, generally used in substance abuse trials, can be adapted and used for pharmacogenetic trials to educate participants on how pharmacogenetics can guide medication management and improve compliance throughout the course of a trial.

### 4.4. Wearable Devices and Digital Biomarkers

Wearable devices have already been used in many studies to capture outcomes and digital biomarkers such as direct measurements of physical activity, 6-minute walk tests, sleep quality, blood pressure, and electrocardiograms [27,28,29]. These have significant implications for various types of pharmacogenetic trials, as wearable devices have already been utilized to capture anxiety episodes, which would also be applicable for psychiatric pharmacogenetic trials [30]. In addition, measuring blood pressure and point-of-care anticoagulation levels could be efficiently implemented for some cardiovascular pharmacogenetic trials. SmokeBeat is an app that uses an individual’s smartwatch to detect smoking by tracking the user’s hand-to-mouth gestures [31]. This has resulted in good accuracy with patient reports, and could be an effective DH tool when testing the utility of pharmacogenetic variants recently implicated in nicotine therapy and smoking cessation [32]. These wearable devices that capture digital biomarkers could further enhance traditional efficacy data collection and lead to improved assessment of medication responses in pharmacogenetic trials. 

### 4.5. Telehealth

Telehealth (also referred to as telemedicine) is the delivery of healthcare services using information and communication technology (generally video conferencing). Although it has been in use for decades as a way to provide healthcare remotely, it increasingly is being utilized across multiple clinical specialties. Moreover, due to the recent COVID-19 pandemic, telehealth has now expanded dramatically as a fundamental element of healthcare service and communication. Using telehealth, clinical services can be provided to underserved populations in a reliable and cost-effective manner when compared to more traditional face-to-face modalities. Some studies have shown telehealth to be effective and satisfactory to patients [33,34,35], and that telehealth has similar health outcomes as face-to-face or telephone delivery of care [36]; however, additional effectiveness studies are needed to confirm patient comprehension. Although not as well established as its role in clinical service, telehealth is emerging as a useful tool when conducting clinical trials [37]. As noted above, telehealth can effectively be used in the clinical trial e-consenting process, but can also be an effective tool to replace in-person visits for subsequent data collection throughout the duration of the trial. 

## 5. Remote Trials

Traditional clinical trials are typically conducted in a medical facility and require regular in-person visits by enrolled patients. However, when using DH technologies, all or part of a clinical trial can be conducted virtually. Clinical trials often have high participant burden, given time and travel required for in-person visits. Completely remote trials can be performed safely and effectively for certain trial types, and some pharmacogenetic trials may actually be ideal for this framework. For example, pharmacogenetic trial study visits can be conducted via telehealth; home nursing companies can be employed to collect patient samples; apps can be used to collect PROs (e.g., quality of life, psychological health, satisfaction with care, social support, etc.), AEs, and drug compliance, as well as deliver real-time dose adjustment information from investigators to patients (or from study teams to blinded investigators); and study medication and supplies can easily be shipped directly to patients. 

**Figure 2:**
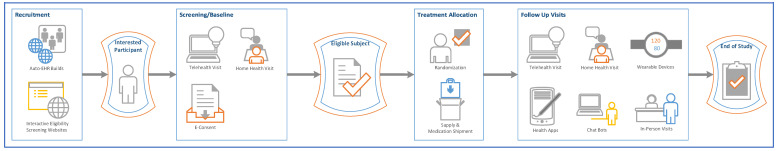
Various digital health technologies can be incorporated to streamline trials; automatic electronic health record (EHR) builds can identify patients, interactive websites can allow patients to enter information directly and if they meet certain criteria can be directed to a chat bot or scheduled for a telehealth visit automatically. Home health visits can be used to collect information that requires additional support/procedures (samples for lab tests, physical exams, administration of medications (i.e., via IV or injection)). Health apps can be used for collecting patient-reported outcomes, adverse events (AEs), concomitant medications, other surveys, and for delivering medication education and dose adjustment information. Reporting of AEs can also be done via a chat bot with an automatic trigger to schedule a telehealth visit if certain criteria are met. Wearable devices can be used to collect vital signs, EKGs, sleep quality, etc. Onsite visits can be included as needed for safety concerns. Some portion of subjects are still expected to be lost to follow up; however DH, technologies may decrease this number.

Limiting onsite visits for patients and building trials around patient homes may increase enrollment, adherence, and retention. One survey study showed patients expressed a preference for using mobile technologies in trials [38]. Figure 2 illustrates a clinical trial model that utilizes multiple DH technologies to conduct a remote trial. A recent study evaluated different trial settings for patients with acute lower-back pain, comparing a decentralized arm via telehealth to a conventional arm via onsite study visits, and a mixed model arm [24]. The study included the following DH technologies: direct data capture, e-consenting, electronic diaries, and a wearable actigraphy patch sensor device. More patients enrolled in the decentralized arm compared to both the conventional and mixed model arms, though sample sizes were small [24]. A small study assessed remote vs. traditional follow-up for cochlear implants for adults, and found that the remote group had an increase in patient activation vs. control group, though quality of life was not different between the groups [39].

Decentralizing trials may also increase enrollment of underrepresented patient populations, thereby increasing diversity in study cohorts, which has been reported as a critical need among pharmacogenetic studies [9]. Pharmacogenetics is known to play a role in alcoholism treatment; however, most studies to date have been conducted in predominantly Caucasian populations [40,41]. Minority patient concerns about participating in clinical trials include psychosocial issues (mistrust, fear lack of confidence), and logistical concerns such as childcare, schedule conflicts, lack of transportation, and appropriate support for understanding consent documents and lack of adequate information about clinical research [42]. Some of these logistic concerns can be addressed by remote trials, as they would involve less travel to study sites, more flexibility in terms of time commitment, and potentially an improvement in communication with the study team through DH apps and telehealth.

## 6. Potential Limitations

While DH has the potential to improve many aspects of clinical trial conduct, there are also some limitations. DH use in trials may skew the age of participants, as younger participants are generally more likely to use DH technologies (e.g., wearable devices, health apps). Similarly, information technology literacy may also confound trials that implement DH technologies, which may also be associated with certain sociodemographic groups and populations with low income. Additionally, if specific apps/interventions are not used to promote engagement during a trial, there can be drop-off in engagement over time, especially in a lengthy trial, which can result in loss of data. Some disease types may not be ideal for using DH technologies in place of onsite study visits, cancer patients for example are frequently required to come to hospitals for their standard of care medical treatment; in these cases, onsite study visits may be more appropriate. Although telehealth offers many logistic advantages when prescribing medications, those with well-characterized safety issues should be administered and monitored onsite by the appropriate care provider. These issues should be carefully considered when designing a trial, and procedures put in place to minimize any negative or confounding effects.

## 7. Conclusions

DH technologies are increasingly being incorporated into clinical trials to facilitate novel trial workflows and outcomes, decrease participant burden, cost-effective operations, and accelerate scientific discovery. Implementing DH applications into pharmacogenetic clinical trials may ultimately lead to increasing patient diversity, improving medication compliance, allowing for double-blinding, and improving delivery of dose adjustment information to both patients and providers. As such, future pharmacogenetic trials could benefit from leveraging DH technologies and trial methodologies in their design and operation.

## References

[1] Bhavnani S.P., Narula J., Sengupta P.P. (2016). Mobile technology and the digitization of healthcare. Eur. Heart J..

[2] Dallery J., Kurti A., Erb P. (2015). A New Frontier: Integrating Behavioral and Digital Technology to Promote Health Behavior. Behav. Anal..

[3] Coran P., Goldsack J.C., Grandinetti C.A., Bakker J.P., Bolognese M., Dorsey E.R., Vasisht K., Amdur A., Dell C., Helfgott J. (2019). Advancing the Use of Mobile Technologies in Clinical Trials: Recommendations from the Clinical Trials Transformation Initiative. Digit. Biomark..

[4] Fabbri C., Zohar J., Serretti A. (2018). Pharmacogenetic tests to guide drug treatment in depression: Comparison of the available testing kits and clinical trials. Prog. Neuropsychopharmacol. Biol. Psychiatry.

[5] Kimmel S.E., French B., Kasner S.E., Johnson J.A., Anderson J.L., Gage B.F., Rosenberg Y.D., Eby C.S., Madigan R.A., McBane R.B. (2013). A pharmacogenetic versus a clinical algorithm for warfarin dosing. N. Engl. J. Med..

[6] Marquis-Gravel G., Roe M.T., Turakhia M.P., Boden W., Temple R., Sharma A., Hirshberg B., Slater P., Craft N., Stockbridge N. (2019). Technology-Enabled Clinical Trials: Transforming Medical Evidence Generation. Circulation.

[7] Huddart R., Sangkuhl K., Whirl-Carrillo M., Klein T.E. (2019). Are Randomized Controlled Trials Necessary to Establish the Value of Implementing Pharmacogenomics in the Clinic?. Clin. Pharmacol. Ther..

[8] De T., Park C.S., Perera M.A. (2019). Cardiovascular Pharmacogenomics: Does It Matter If You’re Black or White?. Annu. Rev. Pharmacol. Toxicol..

[9] Zhang H., De T., Zhong Y., Perera M.A. (2019). The Advantages and Challenges of Diversity in Pharmacogenomics: Can Minority Populations Bring Us Closer to Implementation?. Clin. Pharmacol. Ther..

[10] Levy K.D., Blake K., Fletcher-Hoppe C., Franciosi J., Goto D., Hicks J.K., Holmes A.M., Kanuri S.H., Madden E.B., Musty M.D. (2019). Opportunities to implement a sustainable genomic medicine program: Lessons learned from the IGNITE Network. Genet. Med..

[11] Mullins C.D., Whicher D., Reese E.S., Tunis S. (2010). Generating evidence for comparative effectiveness research using more pragmatic randomized controlled trials. Pharmacoeconomics.

[12] Ford I., Norrie J. (2016). Pragmatic Trials. N. Engl. J. Med..

[13] Fung E., Järvelin M.R., Doshi R.N., Shinbane J.S., Carlson S.K., Grazette L.P., Chang P.M., Sangha R.S., Huikuri H.V., Peters N.S. (2015). Electrocardiographic patch devices and contemporary wireless cardiac monitoring. Front. Physiol..

[14] Arakawa T. (2018). Recent Research and Developing Trends of Wearable Sensors for Detecting Blood Pressure. Sensors.

[15] Choi Y.K., Demiris G., Lin S.Y., Iribarren S.J., Landis C.A., Thompson H.J., McCurry S.M., Heitkemper M.M., Ward T.M. (2018). Smartphone Applications to Support Sleep Self-Management: Review and Evaluation. J. Clin. Sleep Med..

[16] Perlis T.E., Des Jarlais D.C., Friedman S.R., Arasteh K., Turner C.F. (2004). Audio-computerized self-interviewing versus face-to-face interviewing for research data collection at drug abuse treatment programs. Addiction.

[17] Biederman J., Fried R., DiSalvo M., Woodworth K.Y., Biederman I., Noyes E., Faraone S.V., Perlis R.H. (2019). A Novel Text Message Intervention to Improve Adherence to Stimulants in Adults With Attention Deficit/Hyperactivity Disorder. J. Clin. Psychopharmacol..

[18] Fried R., DiSalvo M., Kelberman C., Adler A., McCafferty D., Woodworth K.Y., Green A., Biederman I., Faraone S.V., Biederman J. (2020). An innovative SMS intervention to improve adherence to stimulants in children with ADHD: Preliminary findings. J. Psychopharmacol..

[19] Marsch L.A., Campbell A., Campbell C., Chen C.H., Ertin E., Ghitza U., Lambert-Harris C., Hassanpour S., Holtyn A.F., Hser Y.I. (2020). The application of digital health to the assessment and treatment of substance use disorders: The past, current, and future role of the National Drug Abuse Treatment Clinical Trials Network. J. Subst. Abuse Treat..

[20] Grady C., Cummings S.R., Rowbotham M.C., McConnell M.V., Ashley E.A., Kang G. (2017). Informed Consent. N. Engl. J. Med..

[21] Part 11, Electronic Records; Electronic Signatures—Scope and Application. https://www.fda.gov/regulatory-information/search-fda-guidance-documents/part-11-electronic-records-electronic-signatures-scope-and-application.

[22] Briel M., Olu K.K., von Elm E., Kasenda B., Alturki R., Agarwal A., Bhatnagar N., Schandelmaier S. (2016). A systematic review of discontinued trials suggested that most reasons for recruitment failure were preventable. J. Clin. Epidemiol..

[23] Ni Y., Bermudez M., Kennebeck S., Liddy-Hicks S., Dexheimer J. (2019). A Real-Time Automated Patient Screening System for Clinical Trials Eligibility in an Emergency Department: Design and Evaluation. JMIR Med. Inform..

[24] Sommer C., Zuccolin D., Arnera V., Schmitz N., Adolfsson P., Colombo N., Gilg R., McDowell B. (2018). Building clinical trials around patients: Evaluation and comparison of decentralized and conventional site models in patients with low back pain. Contemp. Clin. Trials Commun..

[25] Jim H.S.L., Hoogland A.I., Brownstein N.C., Barata A., Dicker A.P., Knoop H., Gonzalez B.D., Perkins R., Rollison D., Gilbert S.M. (2020). Innovations in research and clinical care using patient-generated health data. CA Cancer J. Clin..

[26] Torous J., Lipschitz J., Ng M., Firth J. (2020). Dropout rates in clinical trials of smartphone apps for depressive symptoms: A systematic review and meta-analysis. J. Affect. Disord..

[27] Kamišalić A., Fister I., Turkanović M., Karakatič S. (2018). Sensors and Functionalities of Non-Invasive Wrist-Wearable Devices: A Review. Sensors.

[28] Steinberg C., Philippon F., Sanchez M., Fortier-Poisson P., O’Hara G., Molin F., Sarrazin J.F., Nault I., Blier L., Roy K. (2019). A Novel Wearable Device for Continuous Ambulatory ECG Recording: Proof of Concept and Assessment of Signal Quality. Biosensors.

[29] McConnell M.V., Shcherbina A., Pavlovic A., Homburger J.R., Goldfeder R.L., Waggot D., Cho M.K., Rosenberger M.E., Haskell W.L., Myers J. (2017). Feasibility of Obtaining Measures of Lifestyle From a Smartphone App: The MyHeart Counts Cardiovascular Health Study. JAMA Cardiol..

[30] Elgendi M., Menon C. (2019). Assessing Anxiety Disorders Using Wearable Devices: Challenges and Future Directions. Brain Sci..

[31] Dar R. (2018). Effect of Real-Time Monitoring and Notification of Smoking Episodes on Smoking Reduction: A Pilot Study of a Novel Smoking Cessation App. Nicotine Tob Res..

[32] Salloum N.C., Buchalter E.L.F., Chanani S., Espejo G., Ismail M.S., Laine R.O., Nageeb M., Srivastava A.B., Trapp N., Trillo L. (2018). From genes to treatments: A systematic review of the pharmacogenetics in smoking cessation. Pharmacogenomics.

[33] Ekeland A.G., Bowes A., Flottorp S. (2010). Effectiveness of telemedicine: A systematic review of reviews. Int. J. Med. Inform..

[34] Kruse C.S., Krowski N., Rodriguez B., Tran L., Vela J., Brooks M. (2017). Telehealth and patient satisfaction: A systematic review and narrative analysis. BMJ Open.

[35] Powell R.E., Henstenburg J.M., Cooper G., Hollander J.E., Rising K.L. (2017). Patient Perceptions of Telehealth Primary Care Video Visits. Ann. Fam. Med..

[36] Flodgren G., Rachas A., Farmer A.J., Inzitari M., Shepperd S. (2015). Interactive telemedicine: Effects on professional practice and health care outcomes. Cochrane Database Syst. Rev..

[37] Laggis C.W., Williams V.L., Yang X., Kovarik C.L. (2019). Research Techniques Made Simple:Teledermatology in Clinical Trials. J. Investig. Dermatol..

[38] Perry B., Geoghegan C., Lin L., McGuire F.H., Nido V., Grabert B., Morin S.L., Hallinan Z.P., Corneli A. (2019). Patient preferences for using mobile technologies in clinical trials. Contemp. Clin. Trials Commun..

[39] Cullington H., Kitterick P., Weal M., Margol-Gromada M. (2018). Feasibility of personalised remote long-term follow-up of people with cochlear implants: A randomised controlled trial. BMJ Open.

[40] Hartwell E.E., Kranzler H.R. (2019). Pharmacogenetics of alcohol use disorder treatments: An update. Expert. Opin. Drug Metab. Toxicol..

[41] Cservenka A., Yardley M.M., Ray L.A. (2017). Review: Pharmacogenetics of alcoholism treatment: Implications of ethnic diversity. Am. J. Addict..

[42] George S., Duran N., Norris K. (2014). A systematic review of barriers and facilitators to minority research participation among African Americans, Latinos, Asian Americans, and Pacific Islanders. Am. J. Public Health.

